# Oral health-related quality of life and parental anxiety in chinese children undergoing Dental General Anesthesia: a prospective study

**DOI:** 10.1186/s12903-021-01994-2

**Published:** 2021-12-27

**Authors:** Ce Gou, Yan Wang, Ran Yang, Ruijie Huang, Qiong Zhang, Jing Zou

**Affiliations:** State Key Laboratory of Oral Diseases, Department of Pediatric Dentistry, West China Hospital of Stomatology, National Clinical Research Center for Oral Diseases and Sichuan University, Chengdu, China

**Keywords:** Dental General Anaesthesia, Oral health-related quality of life, Parental anxiety, Early Childhood Caries

## Abstract

Dental general anesthesia (DGA) is an effective treatment for very young children or those who have severe dental fear and mental or physical disabilities. However, the long-term impacts of DGA on oral health-related quality of life (OHRQoL) and parents’ anxiety in China are still questionable. This study aimed to assess the influence of DGA on OHRQoL in Chinese children and their parents’ psychological status. A total of 204 children and their parents participated in this study. The ECOHIS was applied to evaluate OHRQoL in children. The parents’ anxiety was analyzed using the Chinese version of the Kessler 10 scale. The internal consistency was evaluated with Cronbach’s alpha coefficient, and Wilcoxon signed-rank test was used for statistical analyses. The scores of each section of ECOHIS decreased one month after the procedure except for the self-image and social sections (*P *< 0.05). The scores of the Kessler 10 scale decreased one month after DGA and kept decreasing six months later (*P *< 0.05). The parents’ Kessler 10 scores exhibited a moderately positive correlation with the children’s ECOHIS scores (r = 0.480, *P *< 0.05). After DGA, the OHRQoL of Chinese children and their parents’ mental health continued to improve. And they exhibited positive correlation.

## Introduction

 Oral health is a multifaceted entity and highly related to the ability to speak, smile, smell, taste, touch, chew, swallow, and convey emotions through facial expressions with confidence and without pain, discomfort, or disease of the craniofacial complex. It reflects the physiological function and status, and psychosocial function essential to the quality of life. It also influences by the person’s changing experiences, perceptions, expectations, and ability to adapt to changing circumstances. The growing literature supports the impact of dental appearance and chronic oral diseases on a person’s quality of life, dental behavior, confidence, self-esteem, family and social relationships, and even career prospects [1]. Oral health-related quality of life (OHRQoL) assesses the impact of oral health problems and covers functional limitations, oral symptoms of dental diseases, and social and emotional well-being [2].

Dental caries, the most prevalent oral chronic disease, manifests as early childhood caries (ECC) in children, remaining a significant public health challenge in Chinese children. Based on the Fourth National Oral Health Epidemiological Survey in mainland China, the prevalence of ECC in children aged 3, 4, and 5 was 50.8%, 63.6%, and 71.9%, respectively, while the constituent ratios of filled teeth were merely 1.5%, 2.9%, and 4.1%, respectively [3]. The severity of dental caries was positively correlated with children’s oral health-related quality of life (OHRQoL), affecting the parents’ emotions and family life [1].

In most circumstances, most children with ECC also accepted dental procedures under non-pharmacological behavior management or sedation strategies. However, for those who are very young or experience severe dental fear and mental or physical disabilities, dental general anesthesia (DGA) is an alternative treatment modality [4]. Compared to the conventional pediatric dentistry strategies, DGA for children is the most effective choice to provide complicated and high-quality dental treatment, recover the morphology and masticatory function, regulate the oral micro-ecological environment, and reduce the caries risk simultaneously in one single visit [5].

Children undergoing a DGA could experience several postoperative complications, which have been reported by previous studies, but the rate of postoperative complications is low. Children reported coughing and pain (27.1%), inability to eat (24.8%), psychological changes (24.1%) and a sore throat (21.1%) 24 h after the DGA [6]. DGA is a kind of day-case anesthesia with a high level of safety and techniques, but parents need to enroll their children for preoperative assessments and learn to cope with the stress of the procedure. Parents of children undergoing DGA are always very anxious about their children’s overall reaction and whether they would regain consciousness. It has been demonstrated that parents’ preoperative anxiety directly impacts the post-anesthetic recovery of the child by transmitting their anxiety to their children and influencing their mood [7]. Meanwhile, the recovery of the child after the operation also affects the anxiety of the parents.

Some published studies conducted in China have showed positive change in OHRQoL following DGA treatment. However, the effects of DGA on OHRQoL and the parents’ anxiety and their correlation have yet to be investigated [8, 9]. Given the paucity of studies on the topic, children with S-ECC following comprehensive dental treatment under general anesthesia in Department of Pediatric Dentistry were selected to assess the impact of DGA on Chinese children’s OHRQoL and their parents’ psychological status and clarify the specific time point of the correlation between parental anxiety and children’s OHRQoL.

## Materials and methods

### Sample selection

Chinese children undergoing DGA from January 2017 to January 2020 in the Department of Pediatric Dentistry were included in this study. This study was conducted by self-paired design with single sample, which contradistinguishing the data longitudinally. Children’s baseline data, including birth date, gender, height, and weight, were collected. The history of systemic diseases and allergies, oral history, and the status of their teeth and dentitions were also obtained before planning DGA [10].

### Inclusion and exclusion criteria

Children were included if they: (1) were between 36 months and 72 months of age; (2) exhibited severe early childhood caries (S-ECC); (3) were classified as class I of American Society of Anesthesiologists Physical Status (ASAP); (4) had never been undergone general anesthesia [10].

Children whose parents or caregivers had a problem in comprehending the medical staff, or rejected postoperative follow-ups, or did not complete the follow-up questionnaire, were excluded [11].

### Procedure of DGA

The general anesthesia protocol was standardized during this study to ensure that the procedure presented no confounding variables. The duration of preoperative fasting and water deprivation was 6 h. Upon entering the operative room, the patients were equipped with standardized monitors by a staff anesthesiologist with >5 years of experience until the general anesthesia ended. Sevoflurane inhalation (3–8% in 2 L/min of oxygen) was used for anesthesia induction. Simultaneously with the disappearance of the eyelash reflex, intravenous access was established, with cisatracurium, propofol and sulfentanyl injected. All the patients accepted nasotracheal intubation. Sevoflurane and propofol were combined to maintain anesthesia. Before the surgery, to avoid the aspiration of secretions and dental materials, a throat gauze pack was used in every patient [1]. Dental procedures were carried out by one certified pediatric dentist and three dental assistants following the Guidelines of the American Academy of Pediatric Dentistry. The tracheal cannula was extubated when the patients’ consciousness, respiratory, swallowing reflex, and cough reflex recovered after the procedure ended. Then, every patient was sent to the post-anesthesia care unit to monitor their health status and recovery. The monitor and oxygen machine were kept operating until the patients achieved the discharge standards. Before discharge, the guardians or caregivers were informed of standardized post-anesthesia advice, and oral hygiene instructions were provided.

### Data collection

The previously validated Chinese version of the Early Childhood Oral Health Impact Scale (ECOHIS) was used to measure the OHRQoL of children under six years of age [12]. It comprises 13 items divided into two sections, i.e., Child Impact Section (CIS) and Family Impact Section (FIS) (Table 1). CIS has four subdomains, including child symptoms (one item), child function (four items), child psychology (two items), and child self-image and social interaction (two items). Parental distress (two items) and family function (two items) are two domains of FIS. Response categories for each question are rated on a five-point Likert scale to record how often an event has occurred during the life of the child relying on the ratings of the scales their parents filled: 0 = never; 1 = hardly ever; 2 = occasionally; 3 = often; 4 = very often; 5 = do not know. The total ECOHIS score ranges from 0 to 52, with the CIS ranging from 0 to 36 and the FIS from 0 to 16. A high ECOHIS score indicates poor oral and dental health and greater oral health impact.

**Table 1:** Selection of ECOHIS before operation (n/%)

Item	Never	hardly ever	Occasionally	Often	very often	don’t know
*CIS*						
Oral pain	26 (12.7)	32 (15.7)	123 (60.3)	21 (10.3)	2 (1.0)	0
Had difficulty drinking hot or cold beverages	74 (36.3)	75 (36.8)	51 (25.0)	4 (2.0)	0	0
Had difficulty eating some foods	34 (16.7)	46 (22.5)	86 (42.2)	32 (15.7)	6 (2.9)	0
Had difficulty pronouncing any words	68 (33.3)	45 (22.1)	59 (28.9)	22 (10.8)	10 (4.9)	0
Missed pre-school, daycare or school	64 (31.4)	59 (28.9)	76 (37.3)	4 (2.0)	1 (0.5)	0
Had trouble sleeping	67 (32.8)	64 (31.4)	67 (32.8)	4 (2.0)	1 (0.5)	1 (0.5)
Been irritable or frustrated	85 (41.7)	79 (38.7)	33 (16.2)	1 (0.5)	1 (0.5)	5 (2.5)
Avoided smiling or laughing	114 (55.9)	63 (30.9)	19 (9.3)	6 (2.9)	2 (1.0)	0
Avoided talking	103 (50.5)	58 (28.4)	34 (16.7)	4 (2.0)	2 (1.0)	3 (1.5)
*FIS*						
Been upset	22 (10.8)	35 (17.2)	102 (50.0)	38 (18.6)	7 (3.4)	0
Felt guilty	5 (2.5)	22 (10.8)	96 (47.1)	66 (32.4)	15 (7.4)	0
Taken time off from work	26 (12.7)	42 (20.6)	120 (58.8)	14 (6.9)	2 (1.0)	0
Had a financial impact on your family	23 (11.3)	56 (27.5)	99 (48.5)	25 (12.3)	1 (0.5)	0

The Kessler 10 scale was initially compiled by Kessler in 1992 to investigate the mental health status of the population [13]. The previously validated Chinese version of the Kessler 10 scale was applied to evaluate the parent’s mental health [14] (Table 2). It assessed the frequency of non-specific psychological symptoms, including anxiety and stress, in the past four weeks. The Kessler 10 scale consists of 10 questions, and response categories for each question are rated on a five-point Likert scale to record how often an event has occurred during the child’s life, relying on the ratings of the scales their parents filled: 1 = never; 2 = seldom; 3 = sometimes; 4 = most of the time; 5 = all the time. The total Kessler 10 score ranges from 10 to 50 points, and a high Kessler 10 score represents a psychological distress.

**Table 2:** Selection of ECOHIS one month after operation (n/%)

Item	Never	hardly ever	Occasionally	Often	very often	don’t know
*CIS*						
Oral pain	64 (31.4)	91 (44.6)	47 (23.0)	1 (0.5)	1 (0.5)	0
Had difficulty drinking hot or cold beverages	86 (42.2)	92 (45.1)	0	1 (0.5)	25 (12.3)	0
Had difficulty eating some foods	75 (36.8)	82 (40.2)	46 (22.5)	1 (0.5)	0	0
Had difficulty pronouncing any words	53 (26.0)	60 (29.4)	64 (31.4)	24 (11.8)	3 (1.5)	0
Missed pre-school, daycare or school	83 (40.7)	86 (42.2)	31 (15.2)	4 (2.0)	0	0
Had trouble sleeping	80 (39.2)	105 (51.5)	19 (9.3)	0	0	0
Been irritable or frustrated	111 (54.4)	86 (42.2)	7 (3.4)	0	0	0
Avoided smiling or laughing	105 (51.5)	75 (36.8)	20 (9.8)	4 (2.0)	0	0
Avoided talking	100 (49.0)	82 (40.2)	19 (9.3)	2 (1.0)	1 (0.5)	0
*FIS*						
Been upset	81 (39.7)	68 (33.3)	47 (23.0)	8 (3.9)	0	0
Felt guilty	65 (31.9)	48 (23.5)	55 (27.0)	32 (15.7)	4 (2.0)	0
Taken time off from work	57 (27.9)	75 (36.8)	64 (31.4)	8 (3.9)	0	0
Had a financial impact on your family	43 (21.1)	67 (32.8)	80 (39.2)	12 (5.9)	2 (1.0)	0

Three visits were included in the regular follow-up agenda, scheduled for each child-parents couple: preoperatively and one and six months after DGA [10]. After the medical staff explained the aims and contents of the questionnaire in detail to the children’s guardians on the day of the procedure, they completed an online questionnaire on the day of DGA and one month and six months after DGA.

### Statistical analysis

Quantitative data analysis of children’s general condition was carried out using SPSS 21.0. The reliability of the ECOHIS and the Kessler 10 questionnaires was analyzed by the internal consistency of Cronbach’s alpha coefficient. As continuous variables were not distributed normally, the preoperative and postoperative scores were compared using the Wilcoxon signed-rank test. The correlation coefficient r was set as the effect size (ES), which demonstrated the magnitude of change. An ES < 0.1 indicated no change, an ES of 0.1– 0.3 meant a small change, an ES of 0.3–0.5 signified moderate change, and an ES of > 0.5 suggested a significant change[8].

The correlation coefficient r was used to analyze the correlation between the ECOHIS and Kessler 10 scores. The absolute value of the correlation coefficient <0.2 indicates very weak or no correlation, 0.2–0.4 demonstrates weak correlation, 0.4–0.6 shows a moderate correlation, 0.6–0.8 indicates a strong correlation, and >0.8 signifies a very strong correlation. The significance level of the test was set at α = 0.05.

## Results

In total, 213 children were recruited for this study at baseline. Three children were not successfully contacted one month after the procedure, and six more children were not successfully contacted during the six-month follow-up. Finally, 204 children participated in this research, with missing values of nine sets and an attrition rate of 4.23%. The missing values were list-wise deleted. The sample comprised 113 male children and 91 female children; the youngest was 36 months old, the oldest was 71 months old, and the average age was 52.78 ± 8.94 months. On average, 16.46 ± 3.26 teeth were treated per child. All the children underwent restorative treatments, and 98.92% of them had endodontic treatment. Tooth extraction was carried out in 57.9% of the children, and 99.7% received stainless steel crowns, with 7.20 ± 1.26 teeth per case.

### Reliability and score analysis of for children

The Cronbach’s alpha coefficients were 0.832, 0.878, and 0.883 before the procedure, one month after the procedure, and six months after the procedure, respectively, indicating the high reliability of the ECOHIS scale.

The “never” options of “Oral pain” and “Avoided smiling or laughing” accounted for 12.7% and 55.9%, respectively, in CIS; therefore, 87.3% of children had oral pain, and only 44.1% of children dared not smile or open their mouths to smile. As for FIS, The least popular option of “never” was “Felt guilty”, while the most popular was “Taken time off from work”, accounting for 2.5% and 12.7% respectively (Table 1).

One month after the procedure, the lowest and highest proportions of “never” options in the CIS report were “Had difficulty pronouncing any words” and “Been irritable or frustrated,” with 26% and 54.4%, respectively. One month after the procedure, 74% of the children exhibited dysphoria, and less than half of the children showed irritability and depression. As for FIS, Only 21.1% of the parents reported they felt no financial pressure (Table 2).

In the CIS report six months after surgery, “Had difficulty pronouncing any words” and “Avoided smiling or laughing” accounted for 32.4% and 56.4% of the “never” choice, respectively. Therefore, six months after the procedure, 67.7% of the children had pronunciation problems, only 43.6% of children did not dare to smile or open their mouth to smile. As for FIS, only a fifth of parents still felt no financial pressure (Table 3).

**Table 3:** Selection of ECOHIS six month after operation (n/%)

Item	Never	hardly ever	Occasionally	Often	very often	don’t know
*CIS*						
Oral pain	80 (39.2)	71 (34.8)	53 (26.0)	0	0	0
Had difficulty drinking hot or cold beverages	97 (47.5)	94 (46.1)	8 (3.9)	3 (1.5)	2 (1.0)	0
Had difficulty eating some foods	85 (41.7)	90 (44.1)	26 (12.7)	2 (1.0)	1 (0.5)	0
Had difficulty pronouncing any words	66 (32.4)	68 (33.3)	45 (22.1)	23 (11.3)	2 (1.0)	0
Missed pre-school, daycare or school	89 (43.6)	93 (45.6)	21 (10.3)	1 (0.5)	0	0
Had trouble sleeping	99 (48.5)	91 (44.6)	11 (5.4)	3 (1.5)	0	0
Been irritable or frustrated	107 (52.5)	89 (43.6)	7 (3.4)	1 (0.5)	0	0
Avoided smiling or laughing	115 (56.4)	66 (32.4)	22 (10.8)	1 (0.5)	0	0
Avoided talking	111 (54.4)	86 (42.2)	4 (2.0)	3 (1.5)	0	0
*FIS*						
Been upset	90 (44.1)	57 (27.9)	55 (27.0)	2 (1.0)	0	0
Felt guilty	57 (27.9)	39 (19.1)	95 (46.6)	11 (5.4)	2 (1.0)	0
Taken time off from work	72 (35.3)	63 (30.9)	66 (32.4)	3 (1.5)	0	0
Had a financial impact on your family	45 (22.1)	83 (40.7)	67 (32.8)	9 (4.4)	0	0

The scores of all the items one month after the procedure were significantly lower than those before the procedure (*P*<0.05), except “Had difficulty pronouncing any words,” “Avoided smiling or laughing,” and “Avoided talking.” Compared with the scores one month after the procedure, the scores of a few items reduced six months after the procedure (*P* <0.05), including “Had difficulty eating some foods,” “Had difficulty pronouncing any words,” “Avoided talking,” and “Had a financial impact,” and the scores of other items and FIS decreased, with no statistically significant difference (*P*>0.05). Except “Had difficulty pronouncing any words,” “Avoided smiling or laughing,” and “Avoided talking,” the ES of each item one month after the procedure was higher than that six months after the procedure (Table 4).

**Table 4:** Scores and ES of each item of ECOHIS [M, (Q25, Q75)]

Item	Before operation	One month after operation	ES of one month after operation	Six month after operation	ES of six month after operation
Oral pain	2 (1,2)	1 (0,1)^a^	0.42	1 (0,2)	0.04
Had difficulty drinking hot or cold beverages	1 (0,2)	1 (0,1)^a^	0.16	1 (0,1)	0.09
Had difficulty eating some foods	2 (1,2)	1 (0,1)^a^	0.40	1 (0,1)^b^	0.11
Had difficulty pronouncing any words	1 (0,2)	1 (0,2)	0	1 (0,2)^b^	0.13
Missed pre-school, daycare or school	1 (0,2)	1 (0,1)^a^	0.22	1 (0,1)	0.08
Had trouble sleeping	1 (0,2)	1 (0,1)^a^	0.25	1 (0,1)	0.09
Been irritable or frustrated	1 (0,1)	0 (0,1)^a^	0.20	0 (0,1)	0.03
Avoided smiling or laughing	0 (0,1)	0 (0,1)	0	0 (0,1)	0.06
Avoided talking	0 (0,1)	1 (0,1)	0.05	0 (0,1)^b^	0.12
Been upset	2(1,2)	1 (0,2)^a^	0.46	1 (0,2)	0.04
Felt guilty	2(2,3)	1 (0,2)^a^	0.45	2 (0,2)	0
Taken time off from work	2(1,2)	1 (0,2)^a^	0.31	1 (0,2)	0.09
Had a financial impact on your family	2(1,2)	1 (1,2)^a^	0.23	1 (1,2)^b^	0.10

^a^The scores of ECOHIS one month after the operation are significantly lower than that before operation (*P* < 0.05)

^b^The scores of ECOHIS six month after the operation are significantly lower than one month after the operation (*P* < 0.05)

The scores of each section, CIS, FIS, and ECOHIS one month after the procedure, obviously reduced compared to the baseline, except the self-image and social sections (*P *< 0.05). The scores of the function section, CIS, family function section, and ECOHIS six months after the procedure decreased compared to the one-month postoperative interval (*P *< 0.05), and the scores of other sections and FIS decreased, which was not significant (*P *> 0.05). One month after the procedure, the ES of each section and total scores were higher than six months after the procedure, except for the family function section (Table 5).

**Table 5:** Scores and ES of each section of ECOHIS [M, (Q25, Q75)]

Section	Before operation	One month after operation	ES of one month after operation	Six month after operation	ES of six month after operation
CIS	10 (6,14)	8 (3,11)^a^	0.34	6 (3,10)^b^	0.14
Symptom	2 (1,2)	1 (0,1)^a^	0.42	1 (0,2)	0.04
Function	5 (3,7)	4 (2,5)^a^	0.28	3 (1,5)^b^	0.16
Psychological	2 (0,3)	1 (0,2)^a^	0.26	1 (0,2)	0.04
Self-image and social	1 (0,2)	1 (0,2)	0	1 (0,2)	0.09
FIS	7 (6,9)	5 (2,7)^a^	0.50	4 (2,7)	0.07
Occupational stress	4 (3,5)	2(0,4)^a^	0.49	2 (0,4)	0
Family function	3 (2,4)	2 (1,3)^a^	0.34	1 (1,2)^b^	0.50
Total scores	17 (12,23)	13 (6,17)^a^	0.46	11 (4,16)^b^	0.16

^a^The scores of ECOHIS one month after the operation are significantly lower than before operation (P <0.05)

^b^The scores of ECOHIS six month after the operation are significantly lower than one month after the operation (P <0.05)

### Reliability and score analysis of Kessler 10 for parents

The Cronbach’s alpha coefficients of the scale before surgery, one month after surgery, and six months after surgery were 0.920, 0.902, and 0.931, respectively, using the internal consistency Cronbach’s alpha coefficient, indicating the high reliability of the Kessler 10 scale. The scores of the Kessler 10 scale for parents one month after the procedure decreased significantly compared with the baseline, with the same trend six months after the procedure compared with one month after the procedure (*P*<0.05). The ES, one month after the procedure, was higher than that six months after the procedure (Table 6).

**Table 6:** Scores and ES of each item of Kessler 10 [M, (Q25, Q75)]

Item	Before operation	One month after operation	ES of one month after operation	Six month after operation	ES of six month after operation
Feel tired out for no good reason	2 (2,3)	2 (1,2)	–	2 (1,2)	–
Feel nervous	2 (2,3)	2 (1,2)	–	2 (1,2)	–
Feel so nervous that nothing could calm you down	1 (1,2)	1 (1,2)	–	1 (1,2)	–
Feel hopeless	2 (1,2)	1 (1,2)	–	1 (1,2)	–
Feel restless or fidgety	2 (1,2)	2 (1,2)	–	2 (1,2)	–
Feel so restless you could not sit still	1 (1,2)	1 (1,2)	–	1 (1,2)	–
Feel depressed	2 (1,2)	1 (1,2)	–	1 (1,2)	–
Feel that everything was an effort	2 (1,2)	1 (1,2)	–	1 (1,2)	–
Feel so sad that nothing could cheer you up	2 (1,2)	2 (1,2)	–	1 (1,2)	–
Feel worthless	1 (1,2)	1 (1,2)	–	1 (1,2)	–
Total scores	18 (13,21)	16 (11,19)^a^	0.21	14 (10,20)^b^	0.10

^a^ The scores of Kessler 10 one month after the operation are significantly lower than before operation (*P* < 0.05)

^b^ The scores of Kessler 10 month after the operation are significantly lower than one month after the operation (*P* < 0.05)

### Correlation analysis between the scores of ECOHIS for children and Kessler 10 for parents

**Fig. 1:**
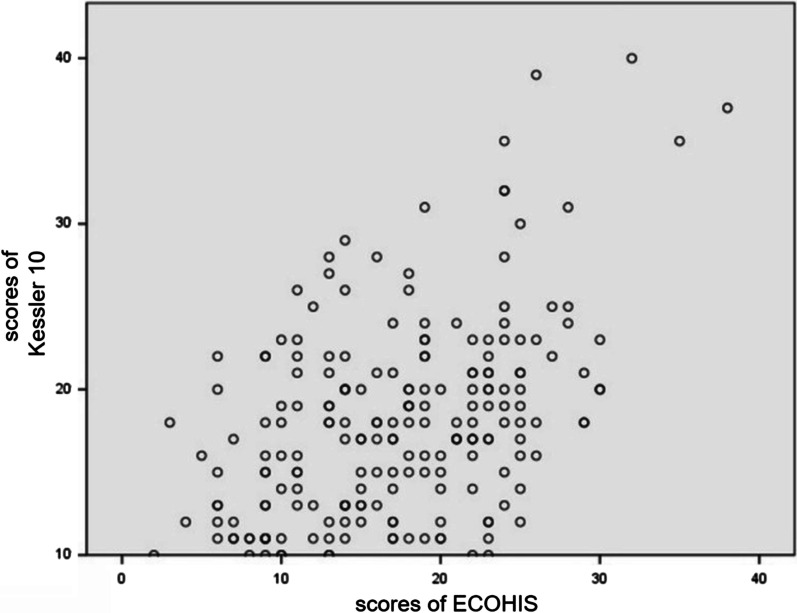
Correlation analysis between the scores of ECOHIS and Kessler 10

After testing, there was a linear relationship between the scores of ECOHIS for children and Kessler 10 for parents, and the correlation coefficient r = 0.480 (*P *< 0.05) suggested a moderate, positive correlation (Fig. 1).

.

## Discussion

A study reported serious parental anxiety when their child underwent dental treatment under day-case anesthesia [7]. Similarly, the psychological problems of the parents affect a child’s quality of life. Markus’ meta-analysis has confirmed the significant relationship between parental and child dental fears, which was most evident in children aged < 8 [15]. When evaluating the effects of treatment for ECC under DGA, we should measure not only children’s OHRQoL but also their parents’ mental health. Therefore, this study evaluated the impact of DGA on the ECOHIS in children with S-ECC and its correlation with their parents’ anxiety by the Kessler-10 scale. The effect size was independent of the magnitude of the research findings, clearly indicating the degree of the relationship between independent and dependent variables. This study used the effect size to evaluate the impact of the dental treatment under general anesthesia on the child’s OHRQoL and the parents’ mental health.

This study compared the ECOHIS scores of children with S-ECC before the DGA and one and six months after the procedure. Before the procedure, “oral pain” exhibited the highest proportion in ECOHIS. Dental caries in children develops quickly, resulting in pain and affecting sleep, eating, and overall growth [16]. It might also cause abscesses, leading to unexplained fever [17]. Postoperative pain might be related to the duration of the DGA procedure or some social and psychological factors [4]. The present study showed that the children’s OHRQoL significantly improved one month after the procedure and continued to improve further during the six-month period after the procedure, consistent with studies abroad [8, 9].

“Had difficulty eating some foods” significantly reduced one month after surgery compared to the baseline; it was statistically significant that it continued to decrease six months after the procedure. Severe caries results in tooth loss, reducing contact surfaces of the antagonist teeth and necessitating a modification of the child’s diet. As reported, pain affects the regulation of glucocorticoids and growth factors, thereby compromising metabolism and nutrition [18]. The occlusal contacts were restored, and the children’s chewing function improved after the procedure, consisting of tooth extractions, root canal treatment, and placing stainless steel crowns (SSC), which brought about further improvements in the next six months.

SSC led to premature contacts and increased occlusal vertical dimension (OVD); therefore, the chewing function was not fully restored. Van der Zee et al. reported that VDO would return to its preoperative state in approximately 30 days in children with 1 to 4 SSCs [19]. The increased OVD can be compensated by the intrusion of the restored tooth and its antagonist, continued re-eruption of other teeth in the arch, or a combination of both. Rose Maria et al. also reported that any changes in occlusion following the SSC placement could settle in four weeks [20]. Increased OVD exposes the periodontal ligament of the restored tooth to increased bite force, resulting in blood flow disturbance in the compressed periodontal ligament (PDL), followed by cell death, called hyalinization, which is resorbed by macrophages, with resorption of the undermined bone through osteoclast activity, eventually leading to tooth movement to settle OVD. Besides, the compressed periodontal ligament discomfort can be a reason for “Had difficulty eating some foods” for children. It might have taken more time to re-establish the new occlusal contacts in this study, with 7 SSCs per child on average.

Furthermore, occlusal bite force (OBF) is an indicator of the masticatory system’s functional status. Owais et al. assessed the functional status of the masticatory system by occlusal bite force (OBF) following the placement of SSC on primary molars and reported that OBF decreased one week after placing PMC restorations and started to increase after one month, reaching its original value after six months[21]. An efficient masticatory function might require a long adaptation time, and parents can train children’s chewing function by selecting proper food[10].

However, the scores of the item “had difficulty pronouncing some words” and the item “avoided talking” exhibited no obvious changes one month after the procedure but decreased after six months. Nearly 90% of all the consonants are produced in the anterior portion of the oral cavity; i.e., the dental arches might be one of the most important factors influencing the production of sounds, as structural boundaries for placing the tongue and lips. A defect in the dental structure or alignment might disturb the normal airflow and pressure and proper lip and tongue placement and contouring, influencing speech sound production integrity. Leavy et al. reported that speech sound production errors were related to the predictive malocclusal traits, and the more severe the malocclusion, the more likely the occurrence of speech sound errors [22]. The vertical height of the posterior tooth increased after restoration with SSC, and the crowned teeth caused supra-occlusion, leading to a transient decrease in the overbite of anterior tooth. The occlusal interferences that led to the instability of the intermaxillary relationship, resulting in masticatory muscle or TMJ tissue damage, could also influence pronunciation[23]. Junichiro et al. called the palate height, the narrowing of the dental arch, and the ratio of front area space “palate and dental arch parameters.” A small front space, large palate height, and narrow dental arch influence speech production [24]. Placing SSCs led to changes in the tongue space, palate height, and dental arch, causing pronunciation problems.

The most serious impact of children’s S-ECC on their parents was guilt before the DGA. One month after the oral procedures, the most frequent item in FIS reports was “had a financial impact on the family.” Six months later, the “financial impact” was still the most reported one. However, the proportion decreased significantly compared with that one month after the procedure. This indicates that DGA exerted more significant financial pressure on the family because the parents had to make a higher one-off payment for the procedure, with little domestic insurance to cover the cost in most families.

China reached near-universal health coverage in a short time, but dental care is mainly excluded from it. In China, health insurance is classified as uninsured, rural resident insurance, urban resident insurance, urban employee insurance, government healthcare program, and otherwise insured [25]. Over 90% of Chinese people possess one of the three major public health insurance plans, known as the New Cooperative Medical Scheme (NCMS), the Urban Resident Basic Medical Insurance (URBMI), and the Urban Employee Basic Medical Insurance (UEBMI). Among them, URBMI covers children and students but with inpatient care only, which means day-case anesthesia is not included. Besides, only basic dental healthcare is included in the benefit packages of three major public health insurance plans, such as extraction of teeth, amalgam restorations, or some low-cost composite resins, and several simple dental surgical procedures. Furthermore, the ceilings of health insurance plans are very low, as are the reimbursement rates. It only covers a small part of the expenditure, and >85% of total dental costs are paid out of the patient’s pocket [26].

American professional oral insurance was developed more than 60 years ago in 1954, most of which is independent of general medical insurance and promotes oral prevention services. However, according to the original China Insurance Regulatory Commission’s official website, there are 24 insurance companies in China with oral insurance products accounting for 13.71% of all insurance companies belonging to commercial insurance [26]. New policies are required to encourage the introduction of commercial dental insurance as a complement to the current basic medical insurance policy and establish a comprehensive insurance system that acknowledges the equity of basic dental care needs and meets specialized demands for advanced dental care. Considering clinical-effectiveness and cost-effectiveness meta-analysis of 19 articles, which demonstrated the effects of preventive measures on decreasing dental caries and financial costs, our government should eliminate the burden of medical treatment expenditures and make the public health insurance cover preventive services for children [27].

The item “avoided smiling and laughing” exhibited no statistically significant change after one month or six months but improved slightly. It is possibly inferred that the children whose confidence and smile were compromised by dental caries were progressively getting rid of the negative impacts. The children also reduced the number of requests for school leave; they should be monitored regularly [8–10].

Anxiety might be defined as concern or tension caused by apprehension of possible misfortune or danger. It is hard to measure and quantify because of its very subjective nature. For example, since parents evaluated their own anxiety through a questionnaire, various factors could have affected the results. Did the parent answer the questions conscientiously? Was the parent able to comprehend the questions, and did the questions mean the same for each person? In this study, the Kessler-10 scale was used to evaluate changes in the parents’ mental health status before and after their children’s DGA. The Kessler-10 scale is a simple measure of psychological distress used as a brief screening tool to identify distress levels. It can assess the population’s mental health status and diagnose psychological disorders such as stress and anxiety [14]. The scale, which is highly valid and reliable, has been translated into many languages, including Chinese, Danish, and French, and is applied worldwide [28].

 A comparison of the K10 scores showed that the parents’ mental health improved significantly one month after the procedure and continued to improve in the next six months. There are some family-related risk factors for ECC, such as parental inability to control the high snacking frequency, parental indifference about the child’s toothbrushing, and toothbrushing frequency less than twice daily, making parents feel guilty because of the lack of attention to their children’s oral health [29]. This was strongly supported in the present study.

Balmer et al. examined the anxiety levels of parents whose children underwent dental general anesthesia before initial assessment, following the assessment, and before GA. It was demonstrated that parental anxiety increased at the initial assessment, and again immediately preoperatively, it continued to increase as the treatment appointment drew near 30]. AlQhtani et al. showed that the parents’ heart rate tended to be the lowest preoperatively (baseline), increasing during the procedure and decreasing ten minutes postoperatively. Compared with being allowed to observe their child in the dental chair, parents had higher oxygen saturation and heart rates when asked to wait outside the operating room, indicating the stress of dental treatment on the parent when they did not observe the child[7]. The parents’ mental health significantly improved one month after the procedure than six months after the surgery, suggesting that as children’s OHRQoL improved rapidly one month after the procedure, the parents’ mental health also improved quickly. One months after the surgery, postoperative discomfort influenced parental satisfaction with the DGA treatment, raising doubts regarding the effect of treatment. Doctor-patient communication and postoperative management of medical staff were crucial at this time, helping parents understand how to prevent and solve postoperative complications. Parents’ satisfaction continued to increase with an improvement in the child’s OHRQoL and a decrease in postoperative discomfort six months after the surgery.

On the other hand, parents’ anxiety can, directly and indirectly, influence their children. There is adequate evidence to support the fact that the parents’ anxiety can be delivered to the child, and parents and children’s upsets are strongly related. When the emotions affect the child, muscular tension, tachycardia, and hyperventilation increase, causing an increase in the person’s sensitivity towards external agents, such as sensitivity to pain, which is significant in dentistry. Parental factors play an essential role in the child’s dental clinic behavior. They should be a partner in the behavior guidance before DGA. Many documents have registered children’s dental fear; however, the effect of parental anxiety on children undergoing DGA has been ignored. Given the paucity of studies on the topic, studies to assess the relationship between children’s OHRQoL and parents’ mental status are of significance.

**Why this paper is important for paediatric dentists**.

- The OHRQoL of children with S-ECC and their parents’ mental health continued to improve after the children underwent DGA.
- The children’s OHRQoL was positively correlated with the parents’ mental status. With an improvement in children’s OHRQoL, the parents’ mental health improved significantly one month after the procedure and continued to improve in the next six months.

## Data Availability

All data generated and analyzed in this study are included within the article or available from the corresponding author on reasonable request.

## Abbreviations

DGA: dental general anesthesia
OHRQoL: oral health-related quality of life
DGA: dental general anesthesia
S-ECC: severe early childhood caries
ASAP: American Society of Anesthesiologists Physical Status
ECOHIS: Early Childhood Oral Health Impact Scale
CIS: Child Impact Section
FIS: Family Impact Section
ES: effect size
SSC: stainless steel crowns
OVD: occlusal vertical dimension
PDL: periodontal ligament
OBF: occlusal bite force
NCMS: New Cooperative Medical Scheme
URBMI: Urban Resident Basic Medical Insurance
UEBMI: Urban Employee Basic Medical Insurance
